# Effects of salinity on photosynthetic characteristics, rhizosphere microbial community structure, and diversity of *Ulmus pumila* L

**DOI:** 10.3389/fpls.2026.1933592

**Published:** 2026-09-16

**Authors:** Kaipeng Jiang, Yuanyuan Niu, Jiwei Zheng, Jingwen Zou, Pu Wang, Yingzhou Tang, Dezong Sui, Yingdan Yuan

**Affiliations:** 1 Jiangsu Academy of Forestry, Nanjing, China; 2 College of Horticulture and Landscape Architecture, Yangzhou University, Yangzhou, China

**Keywords:** growth, photosynthetic characteristics, saline soil, soil microorganisms, *Ulmus pumila*

## Abstract

Salt stress is a key abiotic limiting factor restricting agricultural productivity, making the screening and breeding of salt-tolerant plants essential for alleviating soil salinization. *Ulmus pumila*, a native Chinese tree with fast growth, good wood properties and high tolerance to salt stress and drought, was investigated to reveal its salt adaptation and underlying mechanisms. This study applied phenotypic identification, physiological determination and high-throughput sequencing to analyze growth traits and rhizosphere microbial communities under low, moderate and high salt stress. Our findings revealed that salt stress significantly inhibited seedling height, ground diameter, biomass and other growth indexes in a salinity-dependent manner. Distinct mineral element allocation existed between stems and leaves, with leaves as the main accumulation organ, and *U. pumila* adapted to salt stress by regulating element distribution and maintaining ion homeostasis. Bacterial communities were more sensitive to salt stress than fungi: Proteobacteria increased while Actinobacteria decreased with elevated salinity, and Ascomycota-dominated fungal communities remained structurally stable. Salt stress reshaped microbial co-occurrence networks, with bacteria adapting via species turnover and fungi maintaining function through core taxa. *Ulmus pumila* resists salt stress via integrated strategies of growth regulation, physiological adjustment and rhizosphere microbial synergy, providing a scientific basis for saline-soil vegetation restoration, afforestation and soil sustainable improvement.

## 1 Introduction

Soil salinization is a major abiotic stress factor that limits global sustainable agricultural development and ecological restoration (Feng et al., 2014; Munns, 2002). Salt stress disrupts plant physiological processes through multiple mechanisms, including osmotic stress, ion toxicity, and nutrient imbalance, thereby significantly inhibiting growth and development. This leads to substantial economic losses, such as reduced crop yields and afforestation failures (Herrero and Pérez-Coveta, 2005). According to statistical data from the United Nations Educational, Scientific, and Cultural Organization (UNESCO) and the Food and Agriculture Organization (FAO), saline soils currently cover approximately 950 million hectares globally. Salinization is expected to increasingly compromise arable land productivity in the near future, potentially resulting in a 50% loss by 2050 (Zhang et al., 2013; Wang et al., 2003). Recent studies have further emphasized that improving plant performance on salt-affected lands is important for maintaining agricultural productivity and expanding the utilization of marginal lands (He et al., 2026). In saline soils, salinity stress can inhibit plant growth and development. Therefore, elucidating the molecular and physiological mechanisms underlying plant tolerance to salt stress, together with screening and breeding salt-tolerant germplasm, is important for improving plant resilience and ensuring food security (Zhang et al., 2013; Yuan et al., 2025). These efforts also contribute to the sustainable utilization and ecological restoration of salt-affected lands.

Extensive research has systematically elucidated the complex mechanisms underlying the adverse effects of salt stress on plant growth and physiology. Salinity significantly suppresses plant growth through three concurrent mechanisms: osmotic stress, ion toxicity, and nutrient imbalance (Munns and Tester, 2008). High salinity disrupts the selective permeability of the plasma membrane, causing the efflux of intracellular K^+^ ions and the influx of extracellular Na^+^ ions. This disturbance in ionic homeostasis within the tissues leads to stunted growth and development, ultimately resulting in plant death (Tavakkoli et al., 2011). Salt stress also damages chloroplast structures, reduces chlorophyll content, and decreases the photosynthetic rate (Ma et al., 2011). Sodium chloride (NaCl) inhibits photosynthesis through multiple simultaneous mechanisms, including osmotic stress-induced reductions in water potential and stomatal conductance, feedback inhibition resulting from carbohydrate accumulation, and ion toxicity (e.g., excess Na^+^ and Cl^-^) combined with deficiencies in essential nutrients, such as K^+^ and Ca²^+^, all of which collectively impair photosynthetic activity (Silva et al., 2010).

Most current studies on the effects of salt stress on plant photosynthesis and soil microorganisms have predominantly focused on crops and herbaceous species, with relatively little attention given to woody plants (Gill et al., 2013; Wani et al., 2013). *Ulmus pumila* L. (Siberian elm) has an extensive distribution throughout the northern subtropical and regions of cold temperate climate. As a keystone tree species in East Asia, serving multiple purposes, including timber production, shelter, food, medicine, forage, and ecological protection, *U. pumila* has substantial economic and ecological value (Zhu et al., 2016; Shi et al., 2004; Chen and Xu, 2012; You et al., 2013). Therefore, the creation, evaluation, and screening of salt-tolerant *U. pumila* cultivars are of great significance for afforestation and the sustainable management of coastal saline regions. Under natural field conditions, this study conducted comparative seedling experiments along a NaCl treatment gradient. Through an integrated and systematic analysis of biomass accumulation, photosynthetic performance, and soil microbial communities in *U. pumila*, we quantified and compared the impacts of salt stress on seedling growth and photosynthetic indices, aiming to elucidate the salt tolerance capacity of *U. pumila* seedlings and provide a theoretical and practical foundation for breeding elite cultivars.

This study systematically addressed the following key questions: (1) How do varying salt concentrations affect the phenotypic traits, biomass accumulation, and photosynthetic characteristics of *U. pumila*? (2) What are the distribution and allocation patterns of mineral elements in stems and leaves, and what ion homeostasis regulatory mechanisms does *U. pumila* use under salt stress? (3) How do varying salinity gradients shape the diversity, community structure, and co-occurrence network characteristics of bacteria and fungi in the rhizosphere of *U. pumila*? Based on these questions, we propose the following hypotheses: (1) the impacts of salt stress on the growth parameters and photosynthetic characteristics of *U. pumila* are concentration-dependent, with moderate salt stress potentially promoting the net photosynthetic rate (Pn) by increasing the specific leaf area (SLA), whereas high salt stress strongly suppresses both growth and photosynthetic function. (2) *U. pumila* maintains cellular osmotic and ionic homeostasis to adapt to salt stress by modulating mineral element allocation between stems and leaves and through ion compartmentalization mechanisms, with leaves serving as the primary organs for mineral element accumulation. (3) Bacterial communities exhibit greater responsiveness and sensitivity than fungal communities to salt stress, and salinity gradients exert directional selection on salt-tolerant microbial taxa, with bacteria adapting through high species turnover, whereas fungi maintain community stability via conserved core taxa.

## 2 Materials and methods

### 2.1 Experimental design and sample collection

Healthy *U. pumila* branches were used as experimental materials. Branches with similar growth status and no visible disease or mechanical damage were collected from the same source population/family (F65212) to minimize background variation. Before treatment, the basal ends of the branches were freshly cut and transferred to hydroponic containers for acclimation under uniform conditions.The hydroponic experiment included four salinity treatments: a control treatment (CK, nutrient solution without additional NaCl) and three NaCl treatments, namely low salinity (25 mM NaCl), moderate salinity (50 mM NaCl), and high salinity (75 mM NaCl). Each treatment contained three independent containers as biological replicates, and each container included at least five *U. pumila* branches. The NaCl concentrations were established by dissolving the required amount of NaCl directly into the hydroponic solution (Wang et al., 2022; Jang et al., 2023).

Salt treatment started on April 3, 2025, and ended on September 3, 2025. All containers were maintained under the same light, temperature, humidity, and solution-management conditions. During the treatment period, evaporative water loss was compensated using deionized water, and the solution volume was kept consistent among containers (Wang et al., 2022). At the end of the treatment, branches and leaves were sampled for physiological, elemental, and endophytic microbial analyses. For endophytic microbial sequencing, stem/branch tissues and leaves from branches within the same container were collected and pooled to generate one mixed plant endophyte sample for each biological replicate. In brief, plant tissues were first rinsed with sterile water to remove surface debris and then surface-sterilized using sterilization procedure (75% ethanol and sodium hypochlorite). The success of surface sterilization should be checked by plating the final rinse water on sterile medium or by including a final-rinse control. After surface sterilization, tissues were cut into small pieces with sterile tools, immediately frozen in liquid nitrogen, transported on dry ice, and stored at -80 °C until DNA extraction. Therefore, the microbial data in this study represent endophytic bacterial and fungal communities from mixed branch and leaf tissues (Fan et al., 2023).

### 2.2 Determination of phenotypic, chlorophyll, and biomass parameters of *U. pumila*

Plant growth, biomass, and leaf traits were measured during the peak seedling growth period in August. The abbreviations, units, sampling schemes, and measurement methods for each trait are summarized in **Table 1**. Seedling height was measured using a measuring ruler from the root base (uniformly marked with a marker pen) to the apical bud of the uppermost shoot, with ten randomly selected seedlings measured in each plot (Ta et al., 2025). The ground diameter was determined at a fixed position 2 cm above the root base (previously marked with a marker pen and preferably free of surface irregularities), and this standardized location was used for all subsequent measurements. Measurements were taken using a digital vernier caliper, with two perpendicular readings (rotated by 90°) averaged per seedling, and ten seedlings were measured per plot (Wu et al., 2022). Crown width was assessed by measuring the maximum branch spread in both the north–south and east–west directions using a measuring ruler for each selected experimental plant, with the values recorded and averaged to obtain the crown width. Ten seedlings were measured in each plot (Zhou et al., 2025). The SLA was determined by collecting five fully expanded leaves from each of six spatially distributed positions (north, south, east, west, upper, and lower) on each seedling. The collected leaves were pressed onto millimeter-scale graph paper, and their outlines were carefully traced with a pencil (minimum grid resolution: 1 mm × 1 mm). The number of grid squares occupied by the leaf outline was counted, with squares containing at least half (≥1/2) of the leaf edge counted as one full square and those with less than half (<1/2) excluded. The total number of occupied squares provided an estimate of the leaf area using the grid method. The measured leaves were then oven-dried and weighed, and the SLA was calculated as the leaf area (m²) divided by leaf dry mass (kg). Five seedlings were sampled from each plot (Gunn et al., 1999). Leaf and stem biomass were determined using the whole-weighing method: seedlings were harvested, all leaves were removed, and the fresh weight of the stem was recorded (Poorter et al., 2012). The samples were oven-dried to determine the ratio of dry weight to fresh weight, which was used to calculate the stem and leaf dry weights of the trees (Liu et al., 2025). Chlorophyll content was assessed in ten leaves from three seedlings per experimental plot. Leaves were selected based on the following criteria: mature, functional leaves from lateral branches in the middle portion of the seedling, specifically, the third pair of compound leaves on a lateral branch and the third and fourth leaflets of that compound leaf (counting from the base to the apex). Measurements were performed using a handheld chlorophyll meter (CCM-200) (He et al., 2024; Feng et al., 2025).

**Table 1:** Plant traits, units, sampling schemes, and measurement methods used for Ulmus pumila seedlings.

Trait	Abbreviation	Unit	Measurement method
Seedling height	SH	cm	Measured from the root base to the apical bud using a measuring ruler
Crown width	CW	cm	Maximum branch spread measured in the north–south and east–west directions and averaged
Ground diameter	GD	cm	Measured 2 cm above the root base using a digital vernier caliper; two perpendicular measurements were averaged
Specific leaf area	SLA	m^2^·g^-1^	Leaf area determined using the grid method and divided by oven-dried leaf mass
Chlorophyll content	Chl	mg·g^-1^	Measured using a CCM-200 handheld chlorophyll meter
Biomass	BM	g	Determined using the whole-weighing method followed by dry/fresh mass conversion

### 2.3 Determination of mineral element content

The determinations were conducted during the peak growing season in August. From each plot, three plants were collected and divided into stems and leaves. The samples were packed in kraft paper bags, fixed at 105 °C for 30 minutes, and then dried at 80 °C to constant weight. After drying, the plant materials were ground and sieved prior to further analysis. The powdered samples were digested using an HNO_3_–HClO_4_ mixture, and the concentrations of eight elements (P, Na, K, Ca, Mg, Fe, B, and Mn) were determined by inductively coupled plasma optical emission spectrometry (ICP-MS) (Hansen et al., 2013). The HNO_3_-HClO_4_ digestion procedure was as follows: 0.5 g of powdered sample was treated with 10 mL HNO_3_ and 2 mL HClO_4_ overnight and subsequently heated until the solution became clear. After cooling, the digest was diluted with ultrapure water, filtered, and brought to a final volume of 100 mL. Elemental concentrations were then determined using ICP-MS (Hur et al., 2023).

### 2.4 DNA extraction and high-throughput sequencing of soil microbial communities

Genomic DNA was extracted from 250 mg of each *U. pumila* root-zone soil sample using the MagBeads FastDNA Kit for Soil (116564384) (MP Biomedicals, CA, USA) following the manufacturer’s instructions (Zong et al., 2026). The quality and quantity of the extracted DNA were assessed using 0.8% agarose gel electrophoresis and LitePlus NanoDrop spectrophotometry (Yang et al., 2023). The V5–V7 hypervariable regions of the 16S rRNA gene were amplified by PCR using the primer pair 799F (5′-AACMGGATTAGATACCCKG-3′) and 1193R (5′-ACGTCATCCCsCACCTTCC-3′). For PCR amplification of the fungal ITS region, primers ITS1F (5′- CTTGGTCATTTAGAGGAAGTAA -3′) and ITS2R (5′- GCTGCGTTCTTCATCGATGC -3′) were used (Bates et al., 2011; Yang et al., 2021a). PCR amplification was performed in triplicate, and purified amplicons were sequenced on an Illumina NovaSeq X Plus platform using paired-end 250-bp sequencing (Oliva et al., 2024). Raw reads were quality filtered, merged, and denoised or clustered into ASVs using QIIME2 (Bolyen et al., 2019b). Chimeric sequences, low-abundance artefacts, and non-target sequences were removed. Because the samples were derived from plant tissues, chloroplast, mitochondrial, and host plant-derived sequences were strictly excluded from the bacterial dataset before community composition analyses. Taxonomic assignment was performed against SILVA 138.1 for 16S rRNA and UNITE 8.3 for ITS (Liu et al., 2022b). All samples were rarefied to an equal sequencing depth prior to α- and β-diversity analyses.

### 2.5 Statistical analysis

Photosynthetic data were analyzed using the Data Processing System software. Statistical and variance analyses of the measured data were conducted using SPSS (version 19.0). Mean differences were compared using Duncan’s new multiple range test (*p* ≤ 0.05) (Duncan, 1955). Graphical presentations were created using GraphPad Prism 9 and the R package ggplot2 (version 3.2.0). One-way analysis of variance (ANOVA) was performed to assess the differences in soil properties among the groups (Zhang et al., 2024b). PCA was performed on the standardized bacterial community abundance matrix to visualize variation in bacterial community composition. For the fungal community, PCoA was conducted based on Bray–Curtis dissimilarities to assess differences in community structure among treatments. (version 1.8.0) (Bolyen et al., 2019b).

## 3 Results

### 3.1 Effects of varying salt content in salinity treatments on phenotypic traits and biomass indices of *U.pumila*

The effects of salt stress levels on the phenotypic and biomass parameters of *U. pumila* were examined. Our findings revealed that seedling height, basal diameter, crown width, chlorophyll content, and biomass consistently decreased with increasing salt concentrations, with all control measurements exceeding those recorded under salt stress (**Figures 1a–e**). Notably, seedling height, basal diameter, and crown width differed significantly between high salt stress and both low and medium salt treatments, whereas chlorophyll content and biomass differed significantly between low salt stress and medium and high salt conditions. Conversely, SLA was highest in the CK group, whereas among the NaCl-treated groups, the medium-salt treatment exhibited the highest SLA (**Figure 1f**). These observations likely reflect variations in physiological adaptation strategies that depend on stress intensity.

**Figure 1:**
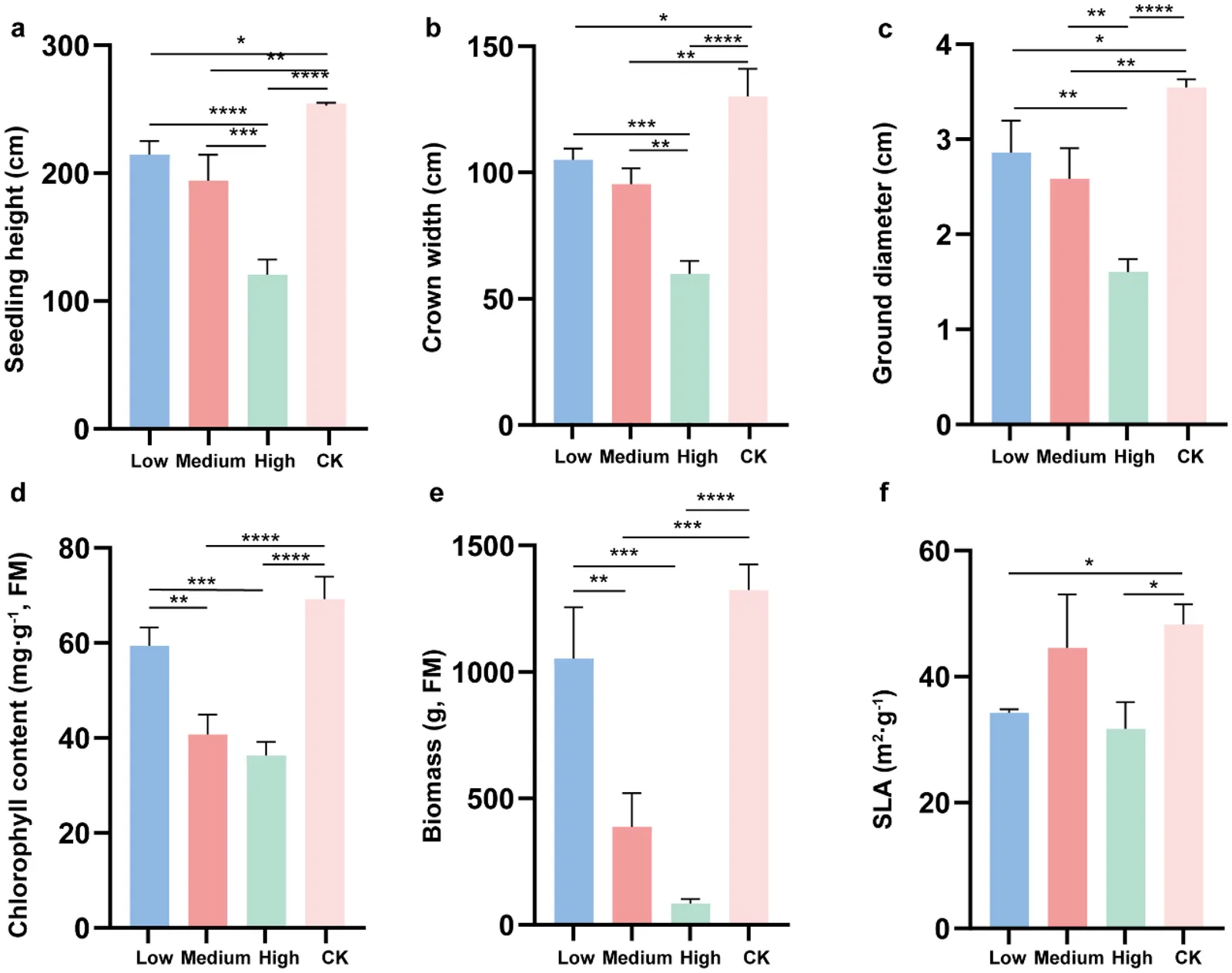
Effects of salinity treatments on phenotypic and biomass parameters of *U. pumila*. **(a)** seedling height, **(b)** crown width, **(c)** basal diameter, **(d)** chlorophyll content, **(e)** biomass, **(f)** SLA. CK, control; low, 25 mM NaCl; medium, 50 mM NaCl; high, 75 mM NaCl. Asterisks indicate significant differences between treatments: *p* < 0.05, *p* < 0.01, *p* < 0.001, and *p* < 0.0001.

### 3.2 Analysis of differences in mineral element content between plant stems and leaves

This study quantified the concentrations of eight essential mineral elements (K, Na, Ca, Mg, Fe, Mn, P, and B) in the stems and leaves of *U. pumila*. The results revealed pronounced and statistically significant differences in elemental distribution between these tissues (**Figure 2**). K concentrations in both stems and leaves initially increased with increasing salt stress but subsequently decreased under high salinity conditions. Inter-treatment differences in K^+^ content were highly significant in both organs (*P* < 0.001; **Figure 2a**). Sodium concentrations remained below 0.5 mg g^-^¹ in stems, whereas leaf Na content exhibited a significant and continuous increase, with highly significant intergroup differences (*P* < 0.001; **Figure 2b**). The Ca content in stems remained relatively stable across treatments, whereas leaf Ca concentrations showed an initial increase, followed by a decline as the stress intensified (**Figure 2c**). The Mg concentrations in all vegetative organs of *U. pumila* followed a consistent upward trend, progressively increasing with increasing stress intensity. ANOVA indicated highly significant inter-treatment differences in Mg²^+^ content across all vegetative organs (*P* < 0.001; **Figure 2d**). Fe concentrations were below 0.5 mg g^-^¹ in stems but increased substantially in leaves, with highly significant intergroup differences (*P* < 0.001; **Figure 2e**). Manganese concentrations increased progressively with salinity in both stems and leaves (**Figure 2f**). Boron exhibited an overall increasing trend with increasing salt concentration, showing significant enrichment compared with the CK group (*P* < 0.001; **Figure 2h**). Collectively, all measured mineral elements accumulated at significantly higher concentrations in leaves than in stems, with most elements exhibiting highly significant inter-treatment differences, indicating that leaves are the primary organs for mineral element accumulation in *U. pumila*.

**Figure 2:**
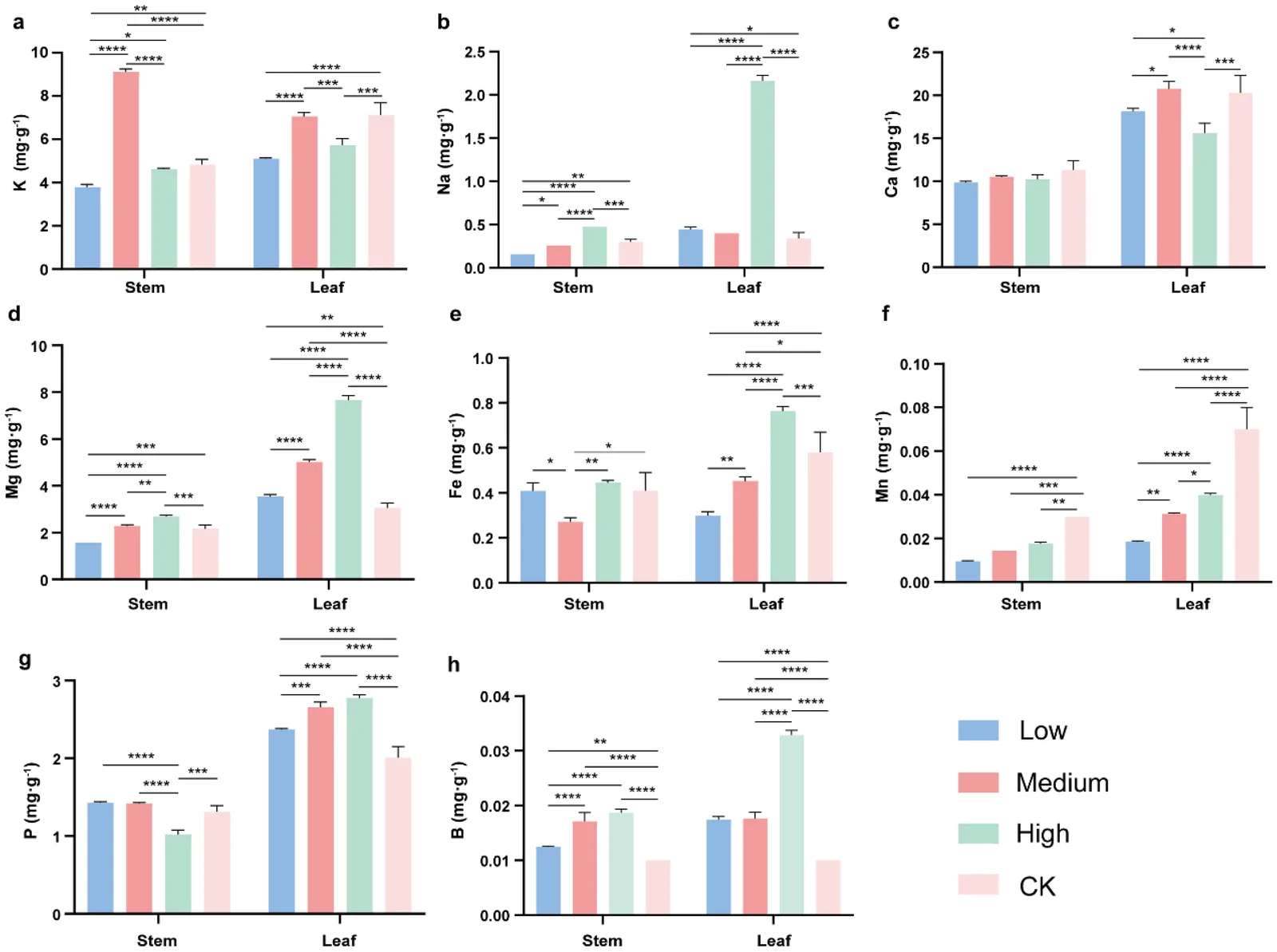
Effects of different salinity treatments on mineral element content in *U. pumila* tissues. **(a)** K, **(b)** Na, **(c)** Ca, **(d)** Mg, **(e)** Fe, **(f)** Mn, **(g)** P, and **(h) **B. CK, control; low, 25 mM NaCl; medium, 50 mM NaCl; high, 75 mM NaCl.

### 3.3 Analysis of soil bacterial and fungal community diversity under different salt stress conditions

This study analyzed the differences in the α-diversity and community structure of soil bacteria and fungi among the low-, medium-, and high-concentration treatment groups and the CK group. The results demonstrated that the different treatments had significant effects on microbial community characteristics (**Figure 3**). Regarding bacterial α-diversity, Pielou’s evenness, richness, and Shannon indices were all significantly higher in the low-salt concentration treatment than in the other groups. Richness increased sequentially with medium and high salt concentrations, reaching a maximum in the CK group, indicating significant intergroup differences (*P* < 0.05; **Figures 3a–c**). For fungal α-diversity, Pielou’s evenness and Shannon indices exhibited similar trends, being lowest in the low-salt treatment and highest in the CK group, with Pielou’s evenness in the medium-salt treatment significantly higher than that in the low-salt treatment (**Figures 3d–f**). Community structure analysis revealed that in the bacterial principal component analysis (PCA), PC1 and PC2 explained 54.13% and 12.91% of the variation, respectively, and ANOSIM indicated highly significant differences between the treatment and CK groups (r = 0.602, *P* = 0.001; **Figure 3g**). In the fungal PCoA, PCoA1 and PCoA2 explained 33.65% and 21.55% of the variation, respectively, with ANOSIM demonstrating highly significant intergroup differences in fungal communities (r = 1.0, *P* = 0.001; **Figure 3h**). Both bacterial and fungal communities from the three treatment groups were distinctly separated from the CK group, and their community composition gradually converged to that of the CK group as the treatment concentration increased.

**Figure 3:**
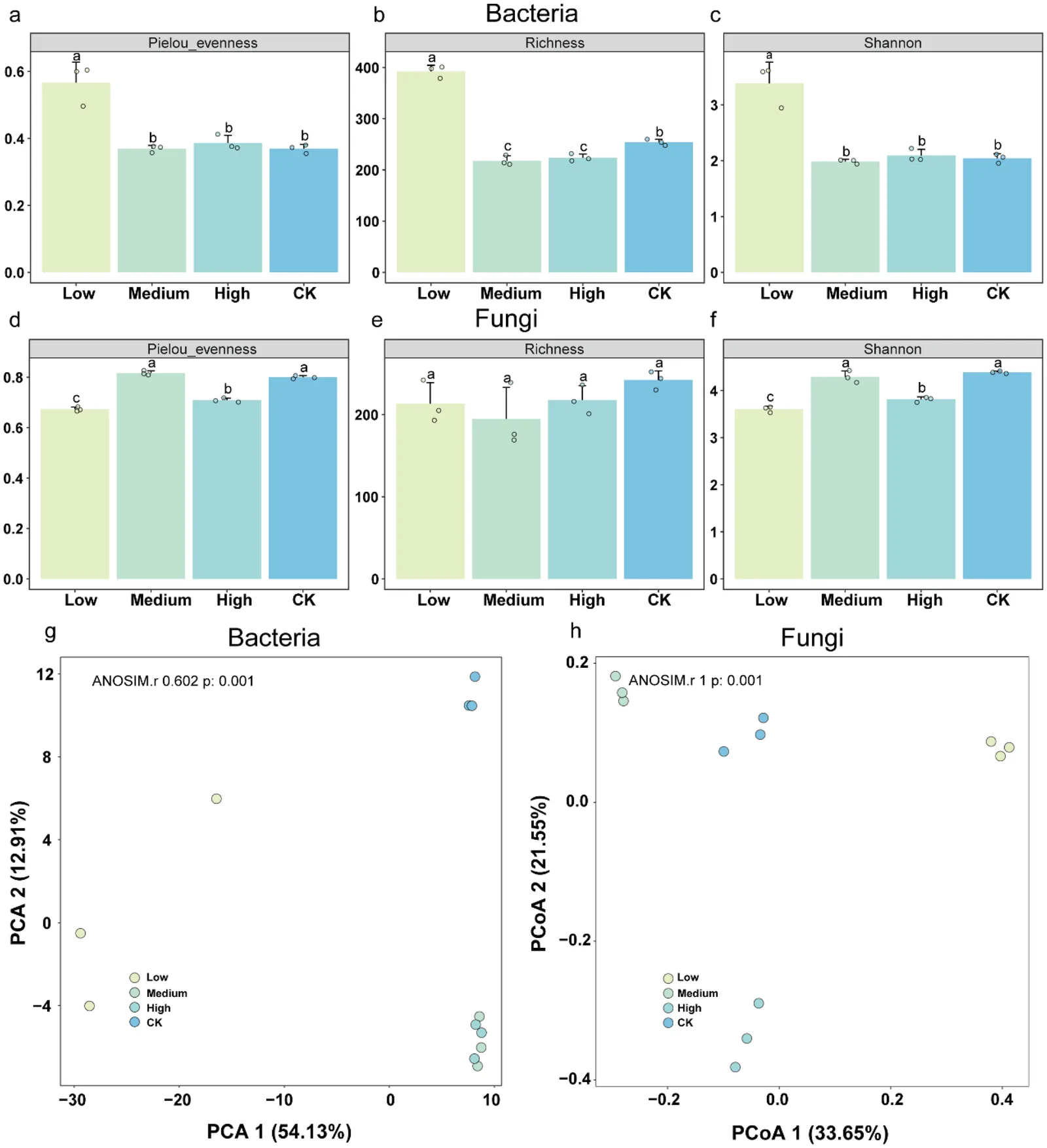
α-diversity and β-diversity of bacteria and fungi in *U. pumila* under different salinity treatments. α-diversity comparisons were performed using ANOVA. **(a–c)** Bacteria, **(d–f)** fungi, **(a, d)** Pielou’s evenness, **(b, e)** richness, and **(c, f)** Shannon, **(g)** PCA, **(h)** PCoA. CK, control; low, 25 mM NaCl; medium, 50 mM NaCl; high, 75 mM NaCl.

### 3.4 Analysis of soil microbial community composition under different salt stress conditions

The composition of microbial communities under varying salt stress concentrations revealed that within the bacterial community, Proteobacteria remained the dominant phylum across all salinity levels, reaching its highest relative abundance at a salt concentration of 50 mM. The relative abundance of Actinobacteria gradually decreased with increasing salt concentrations (**Figure 4a**). As taxonomic resolution became more refined, the divergence between the treatment groups (low, medium, and high) and CK became increasingly pronounced (**Figures 4b–e**). At the genus level, Streptophyta was the dominant community under low-salt conditions, exhibiting the highest relative abundance. Both *Methyloversatilis* and *Aquabacterium* showed minimal relative abundance under low-salt conditions, whereas under medium- and high-salt conditions, their relative abundance approximated those observed in the CK group (**Figure 4e**). Within the fungal community, Ascomycota was the dominant phylum, reaching its maximum relative abundance under medium-salt conditions as salinity increased. Basidiomycota exhibited a decreasing trend in response to elevated salt concentrations (**Figure 4f**). At the genus level, the treated groups exhibited reduced microbial diversity compared with the CK group. *Curvularia* attained its highest relative abundance under medium-salt conditions (**Figure 4j**). Furthermore, the bacterial community composition demonstrated a greater degree of complexity relative to that of the fungal community.

**Figure 4:**
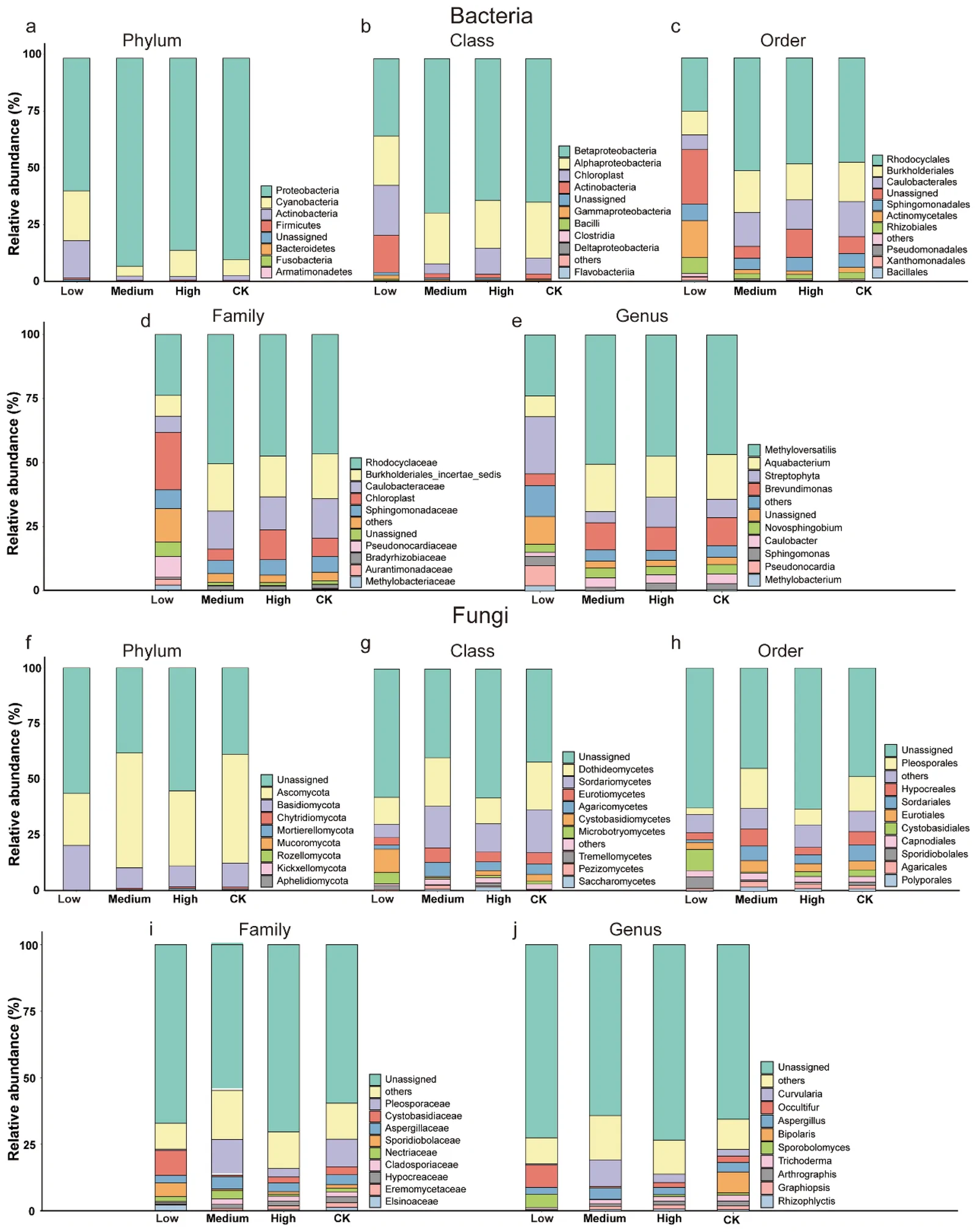
Composition of bacterial and fungal communities associated with *U. pumila* under different salinity treatments. Bacterial communities: **(a)** Phylum, **(b)** class, **(c)** order, **(d)** family, **(e)** genus. Fungal communities: **(f)** phylum, **(g)** class, **(h)** order, **(i)** family, and **(j)** genus. CK, control; low, 25 mM NaCl; medium, 50 mM NaCl; high, 75 mM NaCl.

### 3.5 Effects of salinity gradient on rhizosphere microbial co-occurrence networks

Salinity gradients significantly reshaped the co-occurrence network structure and interaction patterns of rhizosphere bacteria and fungi (**Figures 5a–h**). In bacterial networks, low- and moderate-salinity treatments exhibited multiple densely interconnected blue modules, indicating that positive correlations predominated within the community and strong interspecific cooperative interactions prevailed. Among these, Proteobacteria served as the primary core taxon with high node connectivity, representing keystone species that are critical for maintaining network stability. As salinity increased to high levels, the number of bacterial network modules declined, whereas the proportion of light blue and gray modules increased, reflecting weakened positive interactions, intensified negative associations, and reduced community cooperativity. Concurrently, the node connectivity of Actinobacteria and Firmicutes increased significantly, indicating a salinity-driven shift in the core taxa of the bacterial community under high salt stress. In contrast, fungal co-occurrence networks were consistently dominated by blue modules across all salinity gradients, with only minor changes in module number and connectivity, demonstrating greater resistance and structural stability to salinity stress. Ascomycota persisted as the dominant core taxon across all treatments, maintaining high connectivity even under high-salinity conditions. Its dominant position remained largely unchanged, showing only a slight reduction in the strength of specific module connections, which reflects the adaptive ecological strategy of the fungal community to cope with salt stress by maintaining the stability of the core taxa.

**Figure 5:**
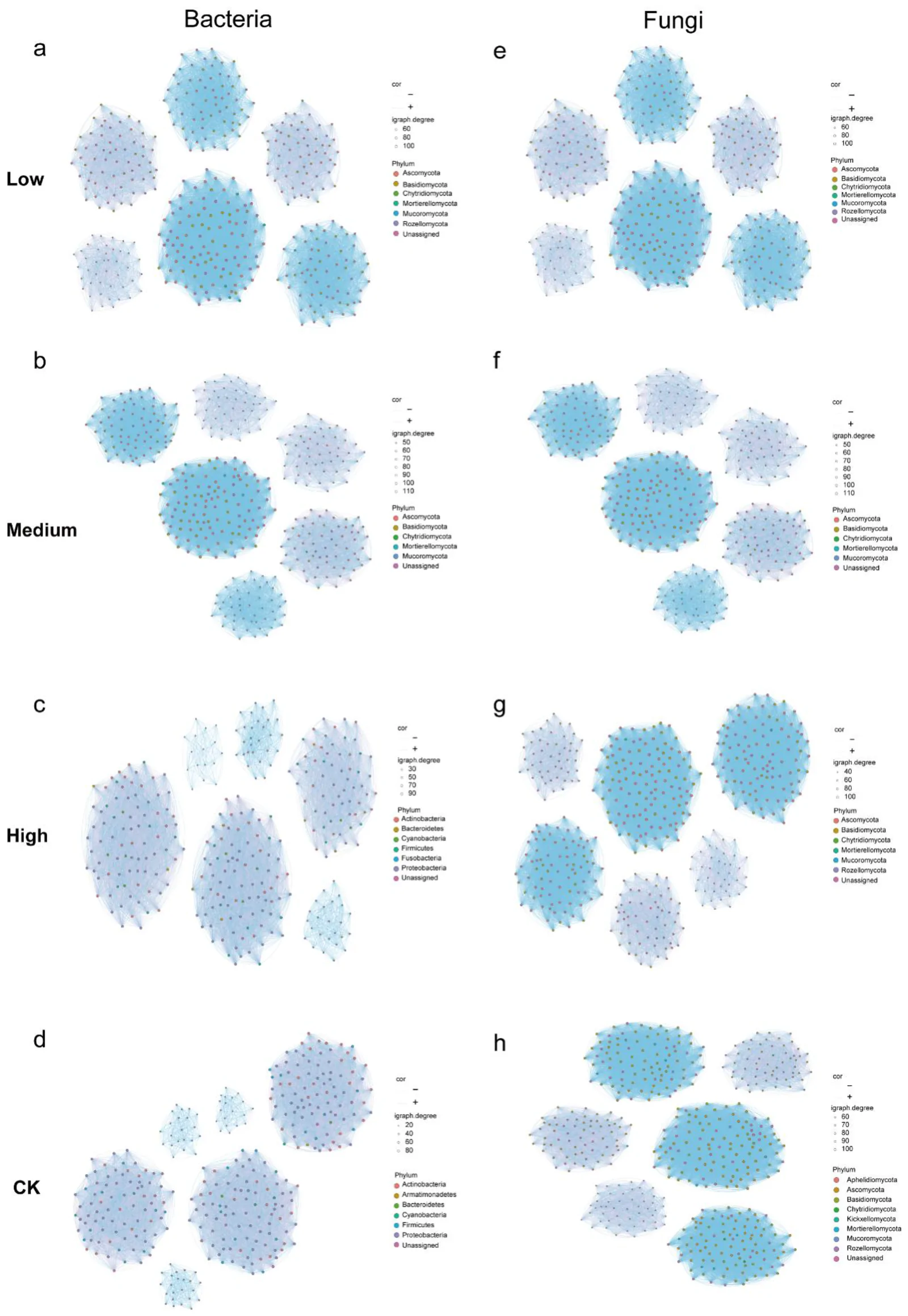
Co-occurrence network characteristics of rhizosphere microorganisms in *U. pumila* across salinity gradients. Node colors correspond to distinct phylum-level taxonomic classifications, and node size denotes species connectivity (larger nodes indicate higher species connectivity). The edge color and thickness represent the nature and strength of interspecies interactions, respectively (blue indicates positive correlations, gray indicates negative correlations, and thicker edges represent stronger correlations). **(a–d)** Bacterial co-occurrence networks; **(e–h)** fungal co-occurrence networks. CK, control; low, 25 mM NaCl; medium, 50 mM NaCl; high, 75 mM NaCl.

### 3.6 Selective effects of salinity gradient on rhizosphere microbial community composition

Salinity gradients exerted strong and significant directional selection on the species composition and relative abundance of rhizosphere bacterial and fungal communities (**Figures 6a, b**). In bacterial communities (**Figure 6a**), as salinity increased progressively from low (A) to high (C), the relative abundances of Betaproteobacteria and Gammaproteobacteria also increased. Notably, Betaproteobacteria were the dominant taxon under high-salinity treatment, exhibiting high within-group consistency in abundance. Conversely, the abundance of Alphaproteobacteria and Chloroflexi declined substantially, whereas Alphaproteobacteria and Actinobacteria showed relatively higher abundance in the CK group. These results demonstrate that increasing salinity significantly enriched salt-tolerant proteobacterial taxa while simultaneously suppressing salt-sensitive groups. In fungal communities (**Figure 6b**), the relative abundance of Cystobasidiomycetes increased sharply under high-salinity conditions, whereas taxa such as Agaricomycetes and Microbotryomycetes exhibited greater relative abundances under low-salinity conditions and in the CK group. Additionally, Saccharomycetes exhibited a relative abundance advantage in medium-salinity treatments, whereas Tremellomycetes maintained relatively stable and moderate abundance across all salinity levels. Collectively, the extent of compositional variation in the dominant fungal taxa was less pronounced than that in bacterial communities.

**Figure 6:**
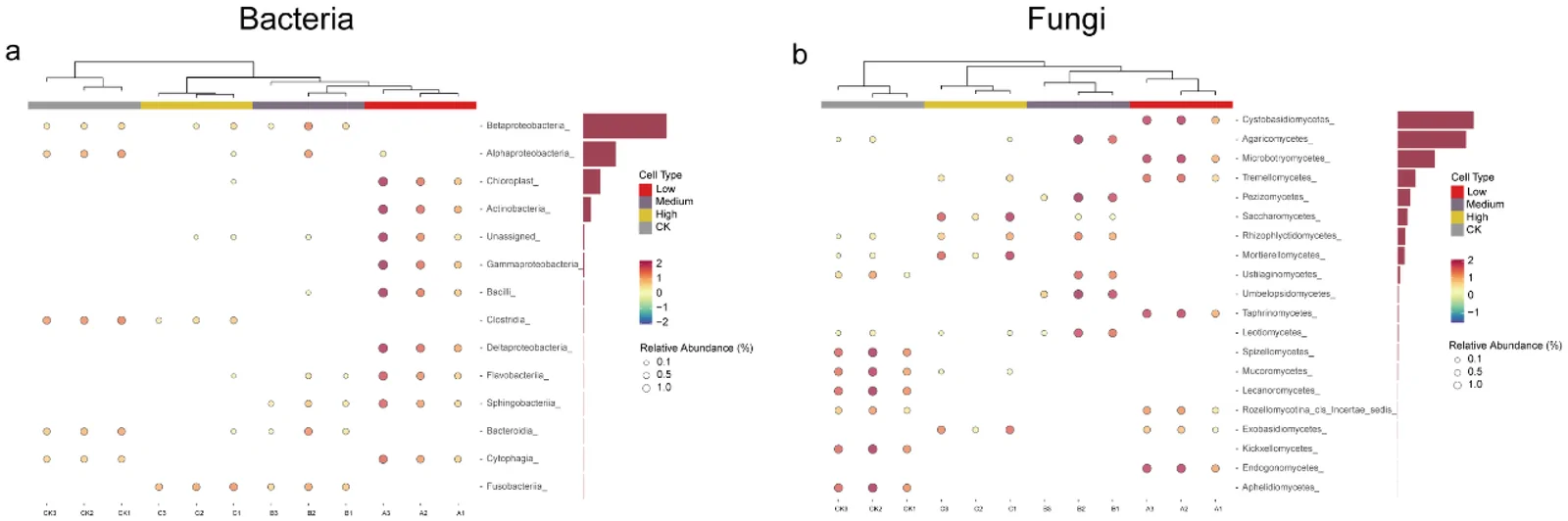
Heatmap of rhizosphere microbial community composition of *U. pumila* under different salinity gradients. Rows represent microbial taxa at the class level, and columns represent samples from different treatments. The dot color and size denote the relative abundance of microbial taxa, with darker colors and larger dots indicating higher relative abundance. The top dendrogram was constructed based on the similarity of community composition among samples, with closer clustering relationships reflecting smaller differences in the microbial community composition between samples. **(a)** Bacteria, **(b)** fungi. CK, control; low, 25 mM NaCl; medium, 50 mM NaCl; high, 75 mM NaCl.

### 3.7 Differential species analysis of rhizosphere microbial communities along a salinity gradient

Pairwise comparisons among the different salinity treatment groups revealed significant differences in the directional selection effects of salinity gradients on rhizosphere bacterial and fungal communities. Bacterial communities exhibited a more pronounced response to salinity changes, with both the number of discriminant taxa and the magnitude of abundance changes increasing significantly as salinity increased from low to high. In the low- versus medium-salinity treatments, genera such as Sphingopyxis and Sphingomonas were significantly enriched under medium-salinity conditions (*P* < 0.05), whereas Novosphingobium and other genera were suppressed. In low- versus high-salinity treatments, the abundance of salt-tolerant bacterial genera, including Hydrogenophaga and Pseudoxanthomonas, increased, becoming the core discriminant species. In medium- versus high-salinity treatments, the abundance of Methylobacillus and other genera further increased, whereas that of *Pseudomonas* and other genera decreased. Compared with the CK, more than 25 bacterial genera showed significant changes in abundance under high-salinity conditions, with Hydrogenophaga exhibiting the greatest increase (**Figures 7a–f**). In contrast, fungal communities showed a more conservative response, with only a few taxa, such as Cystobasidium, Ustilaginales, and Saccharomyces, exhibiting significant differences in abundance across the salinity gradients. The magnitude of change was less than one-fifth of that observed in bacteria, and the number of discriminant taxa was less than half that of bacteria, whereas most taxa remained unchanged (**Figures 8a–f**). These findings indicate that bacteria adapt to salinity changes through rapid species turnover, whereas fungi rely on the stability of core taxa to maintain community function, revealing the distinct ecological strategies used by these two microbial groups in response to salt stress.

**Figure 7:**
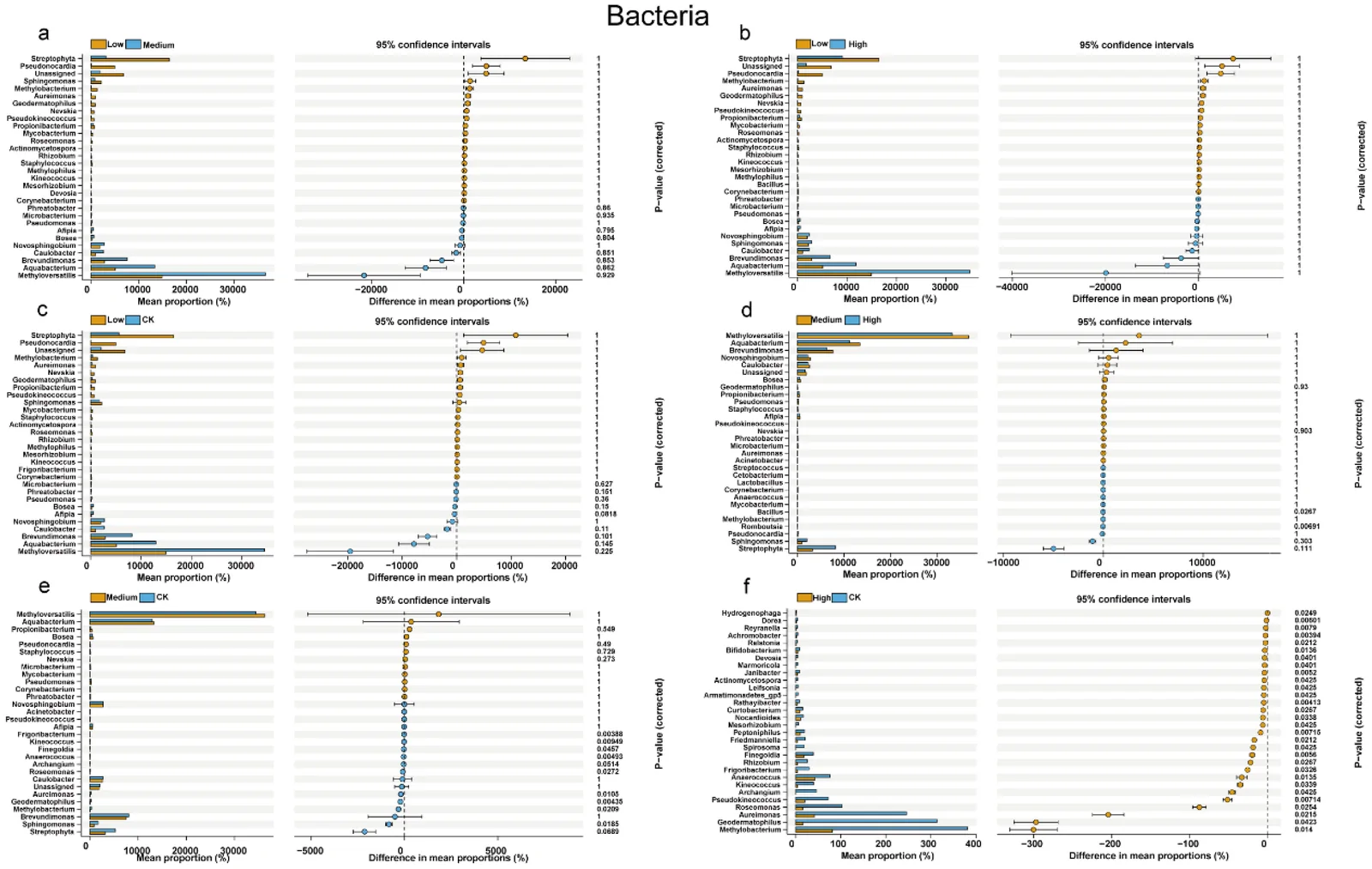
Volcano plot analysis of differential rhizosphere bacterial species across experimental salinity gradients. The panel sequence illustrates pairwise comparative analyses: **(a)** low vs. moderate salinity, **(b)** low vs. high salinity, **(c)** moderate salinity vs. CK, **(d)** moderate salinity vs. high salinity, **(e)** high vs. low salinity, and **(f)** high salinity vs. CK.

**Figure 8:**
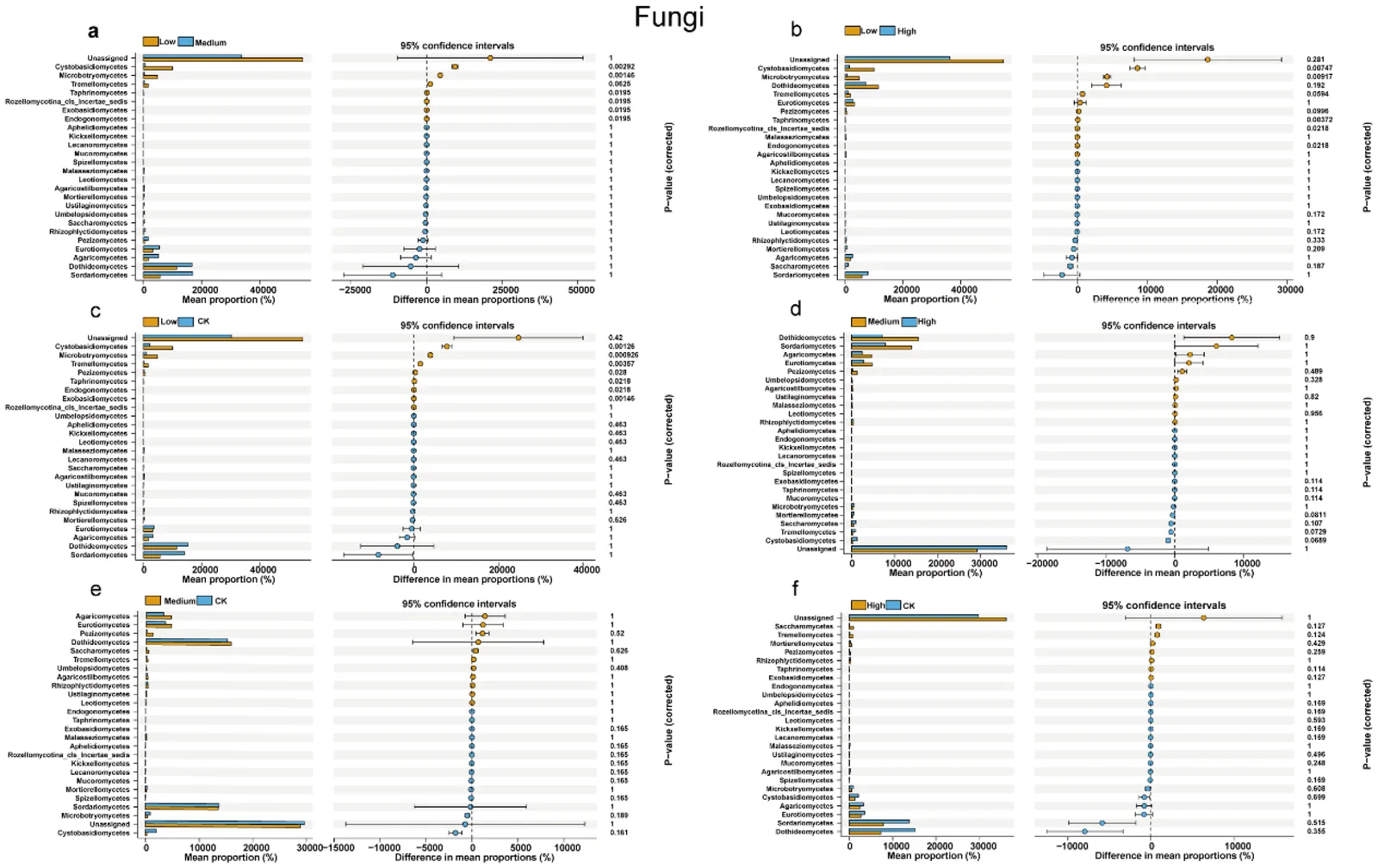
Volcano plot of differentially abundant rhizosphere fungal species across varying salinity gradients. **(a)** Low vs. moderate salinity, **(b)** low vs. high salinity, **(c)** moderate salinity vs. CK, **(d)** moderate salinity vs. high salinity, **(e)** high vs. low salinity, and **(f)** high salinity vs. CK. Significant taxa were identified using one-way ANOVA with FDR-adjusted *p* < 0.05.

## 4 Discussion

### 4.1 Effects of salt stress on growth and photosynthesis in *U. pumila*

Salt stress tolerance originates from multi-level regulatory mechanisms, including the restriction of root absorption of soil salts and their translocation to the shoots, maintenance of ion homeostasis and osmotic balance in root and stem cells, and modulation of leaf development and senescence (Munns, 2005). Plant sensitivity to salt stress is primarily reflected by changes in growth parameters, morphology, and plant physiological functions (Feng et al., 2014). In this study, the growth responses of *U. pumila* varied with salinity intensity. Among the salt-treated groups, seedling height, basal diameter, crown width, chlorophyll content, and total biomass were highest under low salt stress, whereas SLA reached its maximum under moderate salt stress. These patterns indicate that *U. pumila* adjusts its growth and leaf structural traits according to stress intensity. As the NaCl concentration increased to 50 and 75 mmol·L^-^¹, most growth-related parameters declined relative to the low-salt treatment, with biomass showing the most pronounced reduction. Biomass, a key metric for quantifying stress-induced damage and salt tolerance capacity, is widely recognized as a reliable standard for assessing plant adaptation to salinity (Huang et al., 2012). Physiologically, plants optimize resource utilization efficiency and enhance their survival under adverse conditions through strategic adjustments in biomass accumulation and allocation (Grotkopp et al., 2002). This study further confirmed that NaCl treatment significantly suppressed the growth and development of *U. pumila* seedlings, and the inhibitory effect was dependent on the concentration. Specifically, at 25 mmol·L^-^¹ NaCl, plants showed no evident damage symptoms; however, at 50 or 75 mmol·L^-^¹, severe stress symptoms, including leaf chlorosis, necrosis, and eventual abscission, were observed. Collectively, these results indicate that *U. pumila* possesses robust salt stress tolerance, enabling it to maintain normal growth in low-to-moderate salinity environments.

### 4.2 Ionomics of *U. pumila* under salt stress

Soil salinization severely limit agricultural productivity. However, the mechanistic basis of salt stress responses in *U. pumila* remains poorly understood (Guo et al., 2022). This study used ionomics to investigate the effects of salt stress on the stems and leaves of *U. pumila.* Our findings revealed that salt stress significantly inhibited *U. pumila* growth, likely due to high Na^+^ concentrations, which induce osmotic imbalance, membrane dysfunction, and elevated reactive oxygen species production. These factors collectively disrupt plant nutrient metabolism, impairing cell division and elongation (Bracci et al., 2008; Lokhande et al., 2011; Yang and Guo, 2018). Salt stress not only suppresses nutrient acquisition but also alters the translocation of specific minerals. Analysis of mineral element concentrations in *U. pumila* stems and leaves demonstrated that salt stress enhanced the uptake and transport of Na, Mg, Mn, and B in stems, as well as Na, Mg, Fe, Mn, P, and B in leaves, while inhibiting P absorption in stems. To cope with salt stress, *U. pumila* accumulated substantial Na^+^ and restricted vacuolar accumulation via ion compartmentalization. This strategy facilitates cellular water uptake, mitigates the toxic effects of salt ions on cell membranes and cytosolic enzymes, and preserves osmotic homeostasis to ensure normal physiological functions (Graus et al., 2018). Nevertheless, Ca²^+^ exhibited antagonistic interactions with Na^+^, and excessive Na^+^ uptake precipitated Ca²^+^ deficiency in *U. pumila* (Song et al., 2025). Our results further corroborate this inverse relationship, demonstrating that Ca²^+^ content declines concomitantly with increasing Na^+^ accumulation. P is an essential macronutrient for plant growth and plays a fundamental role in seedling vigor and development. In this study, the P concentration in *U. pumila* stems decreased progressively with increasing salt concentrations, whereas the foliar P content correspondingly increased. This contrasting allocation pattern likely reflects the pivotal role of P in photophosphorylation, ATP synthesis, and nucleic acid metabolism, with leaves serving as the primary sites of photosynthesis (Yan et al., 2024). Under salt stress, plants must maintain efficient carbon assimilation to sustain their energy supply (Thomas et al., 2006). Consequently, as external salinity increases, *U. pumila* likely upregulates the expression of phosphate transporters (e.g., members of the PHT family) to preferentially redistribute root-absorbed and stem-reserved P to its leaves. Meanwhile, P accumulation in stems is reduced as a result of this “diversion” effect, given that stems primarily function in structural support and long-distance transport rather than metabolic assimilation (Friji et al., 2025; Sun et al., 2018). In summary, *U. pumila* establishes a coordinated and systematic salt stress adaptation mechanism through the integrated regulation of mineral element partitioning, enhanced ion sequestration capacity, and maintenance of the Ca²^+^–Na^+^ antagonistic balance, thereby providing a theoretical basis for breeding salt-tolerant forest tree species.

### 4.3 Soil microbial community structure of *U. pumila* in coastal saline soils

Similar to the human gut microbiome, rhizosphere microorganisms are considered the second or extended microbiome of plant hosts. These microorganisms play crucial roles in facilitating the uptake and transport of soil nutrients, enhancing plant immunity, and improving plant tolerance to biotic and abiotic stresses (Zhang et al., 2024b). Investigating the composition of rhizosphere microbial communities and identifying key microorganisms involved in salinity stress tolerance in saline soils can significantly contribute to the advancement of agriculture in challenging environments (Krishnamoorthy et al., 2016; Xu et al., 2022). This study used high-throughput sequencing technology to examine the diversity of rhizosphere soil microorganisms associated with *U. pumila* in the coastal saline soils of Jiangsu Province under varying salinity conditions. The rhizosphere fungal communities were primarily composed of Ascomycota and Basidiomycota at different salinity levels. Consistent with multiple reports, Ascomycota and Basidiomycota were the dominant fungal groups in coastal saline soils (Liu et al., 2023a; Yang et al., 2021a). Among soil fungi, Ascomycota is the predominant phylum, with most genera being saprotrophic microorganisms that play vital roles in soil organic matter degradation (Xiao et al., 2024). Ascomycota is the most dominant fungal phylum in coastal areas, whereas Basidiomycota is predominant in inland regions (Liu et al., 2022a). Therefore, in this study, Ascomycota demonstrated adaptability to diverse saline environments, whereas Basidiomycota were dominant only under specific saline conditions. At the genus level, the relative abundance of *Curvularia* generally increased under different salinity conditions compared to that in the CK group, whereas the abundance of *Bipolaris* showed little change. The increased relative abundance of *Curvularia* under salinity treatments suggests that this genus may be associated with saline conditions; however, its potential role in host salt tolerance requires further functional validation. Previous studies have reported that bacterial communities in saline soil samples predominantly belong to Proteobacteria, Actinobacteria, Firmicutes, Bacteroidetes, Cyanobacteria, Blastomonas, Gemmatimonadetes, Acidobacteria, Thermomicrobia, Planctomycetes, and Chloroflexi (Bingqian et al., 2019). In this study, the bacterial communities in salt-affected soils were mainly composed of Proteobacteria and Cyanobacteria. In addition, Proteobacteria, Bacteroidota, and Acidobacteria were significantly correlated with EC values (Liu et al., 2020). With increasing salinity, the relative abundance of Proteobacteria increased, whereas that of Actinobacteria gradually decreased. This aligns with previous studies on bacterial diversity, which demonstrated that Proteobacteria had the highest abundance (>38%) in wetlands and was positively correlated with soil salinity (Chi et al., 2021; Liu et al., 2023b; Yang et al., 2021a; Zhang et al., 2024b). At the genus level, Methyloversatilis, Aquabacterium, and Brevundimonas showed increasing trends with increasing salt concentrations. Therefore, these genera may be associated with the adaptation of *U. pumila* to high salinity stress.

### 4.4 Ecological significance of salinity gradients in rhizosphere microbial co-occurrence networks

This study revealed that salinity gradients exert differential and significant effects on bacterial and fungal co-occurrence networks, revealing distinct ecological adaptation strategies used by these two microbial groups in response to salt stress. Bacterial networks exhibited tightly coupled positive interactions under low and moderate salinities, reflecting enhanced resource utilization efficiency and improved environmental adaptability through collaborative relationships within the communities. However, under high salt stress, these positive interactions weakened, and keystone species were replaced, with a compositional shift from Proteobacteria to Actinobacteria and Firmicutes. This observation aligns with previous studies that concluded that “salt stress disrupts established microbial cooperative relationships and selects for salt-tolerant dominant taxa” (Zhang et al., 2023). Actinobacterial and Firmicute species typically possess superior osmoregulatory capacities and enhanced extracellular enzyme secretion, and their emergence as core taxa under high salinity contributes to maintaining community functional stability. In contrast, fungal co-occurrence networks remained highly stable across all salinity gradients, with Ascomycota consistently serving as the core phylum, likely owing to the ubiquitous presence of thick-walled spores, osmoregulatory genes, and versatile metabolic capabilities in ascomycetes (Zhang et al., 2024a). The robust stability of fungal networks not only preserves community structure but may also indirectly mitigate the negative effects of salt stress on rhizosphere microecology through cross-kingdom interactions with bacteria. These divergent network responses further underscore the functional complementarity of rhizosphere microbial communities in coping with salt stress: bacteria respond rapidly to environmental fluctuations through dynamic restructuring of network topology and keystone species, whereas fungi provide ecological buffering via their stable network architecture. This complementary mechanism may provide a critical foundation for sustaining rhizosphere microecological functions in saline soils and offers theoretical support for enhancing plant salt tolerance through microbial modulation strategies.

## 5 Conclusion

This study shows that NaCl salinity gradients suppressed growth of *U. pumila* seedlings while inducing coordinated changes in leaf structure, ion allocation, and rhizosphere microbial composition. The most distinct plant-level response was organ-specific mineral allocation, with leaves acting as major sinks for Na and several nutrient elements. The microbial response was characterized by stronger bacterial turnover than fungal turnover, with Proteobacteria increasing and Ascomycota-dominated fungal communities remaining comparatively stable across salinity levels. These findings advance understanding of how a salt-tolerant woody species responds to saline conditions at the plant trait, ionomic, and rhizosphere community levels. For application, *U. pumila* remains a promising species for afforestation in saline soils, but the microbial taxa identified here should be regarded as candidates for future functional validation rather than proven drivers of salt tolerance.

## Data Availability

The datasets presented in this study can be found in online repositories, accession number PRJNA1511579.
